# Associations of carotid atherosclerosis with cognitive function and brain health: Findings from a UK tri-ethnic cohort study (Southall and Brent Revisited)

**DOI:** 10.1016/j.athplu.2024.01.002

**Published:** 2024-01-30

**Authors:** Rayan Anbar, Siana Jones, Nish Chaturvedi, Carole Sudre, Marcus Richards, Salahaden R. Sultan, Alun D. Hughes

**Affiliations:** a MRC unit for Lifelong Health & Ageing, Department of Population Science & Experimental Medicine, Institute of Cardiovascular Science, University College London, London, UK; b Department of Radiologic Sciences, Faculty of Applied Medical Sciences, King AbdulAziz University, Jeddah, Saudi Arabia

**Keywords:** Atherosclerosis, Carotid artery, Cognitive function, Intima-media thickness, White matter hyperintensity, Brain atrophy

## Abstract

**Background:**

Cognitive function has an important role in determining the quality of life of older adults. Cardiovascular disease (CVD) is common in older people and may compromise cognitive performance; however, the extent to which this is related to carotid atherosclerosis is unclear.

**Aim:**

We investigated associations between carotid atherosclerosis and cognitive function and neuroimaging markers of brain health in a UK multi-ethnic community-based sample including older people of European, South Asian, and African-Caribbean ethnicity.

**Methods:**

Carotid plaques and intima-media thickness (cIMT) were assessed using ultrasound in 985 people (mean age 73.2y, 56 % male). Associations of carotid atherosclerosis with cognitive function (memory, executive function, language and CSI-D, a global measure of cognitive state) and neuroimaging measures (total brain volume, hippocampal volume, white matter (WM) lesion volume and coalescence score) were analysed using regression analyses, with and without adjustment for potential confounders using two models: 1) adjustment for age, sex, and ethnicity; 2) model 1 plus education, physical activity category, body mass index, hypertension, diabetes, total and high density lipoprotein cholesterol, atrial fibrillation, smoking, previous CVD, alcohol consumption, and presence of chronic kidney disease.

**Results:**

People with carotid plaque or higher cIMT had lower CSI-D score, poorer memory poorer executive function and higher WM lesion volume and coalescence. Language was poorer in people with plaque but was not correlated with cIMT. Associations with plaque were preserved after full adjustment (model 2) but relationships for cIMT were attenuated. Associations with other plaque characteristics were generally unconvincing after adjustment.

**Conclusions:**

This multi-ethnic cohort study provides evidence that presence of carotid plaque, is associated with poorer cognitive function and brain health.

## Highlights

Presence:
• Strong association with lower global cognitive function (CSI-D) in older adults.
• Memory and Executive Function:
• Carotid plaque linked to poorer memory and executive function in a multi-ethnic sample.
• Neuroimaging Markers:
• Presence of carotid plaque associated with higher white matter lesion volume.
• No clear correlation between carotid intima-media thickness (cIMT) and cognitive outcomes.
• Associations between carotid plaque and cognitive decline maintained after extensive adjustments for potential confounders.

## 1 Introduction

Cognitive function has an important role in determining the quality of life of older adults and impaired cognitive function and dementias are major public health concerns [1]. Cardiovascular disease (CVD) is common in older people and influences cognitive performance. For example, vascular dementia is the second most commonly diagnosed type of dementia after Alzheimer's disease in the elderly[2]. Two recent systematic reviews have examined the relationship between carotid atherosclerosis and cognition. Baradaran et al. [3] found evidence of an association between carotid artery plaque and poorer cognition based on clinical diagnosis or cognitive testing, while we [4] found some limited evidence for an adverse association between carotid atherosclerosis and a quantitative measure of cognitive performance, the Mini-Mental State Examination (MMSE). Neither review was able to account fully for confounding due to the disparate nature of the studies included. Moreover, there is inconsistent information about the association between carotid atherosclerosis and particular cognitive domains or neuroimaging markers of brain health, although associations between CVD and poorer performance in tests of memory, attention, processing speed and executive function have been reported[5,6]

While carotid atherosclerosis is a well-recognised risk factor for ischaemic stroke [7], previous studies have also reported associations between carotid atherosclerosis [8,9] or carotid stenosis [10] in the absence of stroke and neuroimaging markers of brain health, such as atrophy or the presence of white matter hyperintensity (WMH). The extent to which these associations reflect the direct consequences of carotid atherosclerotic disease as opposed to concomitant systemic atherosclerosis, cerebral small vessel disease, or some other process that accompanies carotid atherosclerosis remains unclear[9,11]. Understanding these relationships may be important for understanding mechanisms and treatments, as some studies have reported that executive function, visuospatial episodic memory, and psychomotor speed improve after revascularization of carotid arteries[12,13]. Another limitation of current evidence is that most investigations of atherosclerosis and cognitive function or brain health have been performed in European-origin populations. Studies in multi-ethnic populations have been small and infrequent [[14], [15], [16]]. Ethnic differences in susceptibility to cardiovascular disease are well-recognised [[17], [18], [19]], and vascular risk factors for dementia may not be generalizable to different ethnic groups[17].

The aim of this study therefore was to investigate cross-sectional associations between carotid atherosclerosis and cognitive function and brain health in a UK multi-ethnic community-based sample including people of European, South Asian, and African-Caribbean ethnicity.

## 2 Methods

### 2.1 Participants and study design

Detailed information about the Southall and Brent Revisited study (SABRE) has been published previously [20,21]. SABRE was set up in 1988 as a tri-ethnic longitudinal cohort study to examine ethnic differences in chronic disease. It recruited predominantly European (EA), South Asian (SA) and African Caribbean (AC) participants living in West and North London; details have been previously described [20]. Approval for the current study was obtained from the NRES Committee London, Fulham (ref. 14/LO/0108).

For this study, we used data on 985 participants, who attended for clinical investigations (visit 3) over the period 2014 to 2018. All participants completed a health and lifestyle questionnaire [21], where information was recorded on age, sex, health, medical history, and medication. Participants’ ethnicity was determined by interviewers based on grandparental origin and confirmed by participants. All participants were asked to refrain from alcohol, smoking, and caffeine for ≥12 h before clinic attendance. Height and weight were measured using a standardized protocol, body mass index (BMI) was calculated using a Tanita BC 418 body composition analyzer. Seated brachial blood pressure (BP) was measured according to ESH guidelines[22]. The average of the second and third recordings was used as clinic BP. Alcohol consumption was categorised according to UK guidelines into none, ≤14 units per week or >14 units per week. Physical activity estimated as the total weekly energy (MJ) expended in sporting activities, cycling, walking, and in other strenuous activity during leisure time was categorised based on ethnicity-appropriate criteria [23] into: low (<1.5 kcal/kg/day), moderate (≥1.5 kcal/kg/day to <3 kcal/kg/day), moderate to high (≥3 kcal/kg/day to <6 kcal/kg/day) and high (≥6 kcal/kg/day).

### 2.2 Carotid ultrasound assessment

Carotid ultrasound scans were performed by an experienced sonographer as described in detail previously [21], [24] using a protocol based on [26]. Scan were performed with a GE Vivid I Ultrasound system equipped with a 6–13 MHz broadband linear array transducer (12L-RS) along with a 3-lead ECG recording. The common carotid artery (CCA), internal carotid artery (ICA) and external carotid artery (ECA) were imaged bilaterally and three-angle longitudinal views (lateral, posterior, and anterior) plus transverse views were saved as cine loops of 3–5 cardiac cycles. In addition to 2D-images, spectral-Doppler imaging, Colour and Power doppler were recorded and used to assist with plaque identification and delineation. Quantitative analyses were performed offline using validated software (AMS II) that allowed automated measurement of cIMT, plaque area, categorisation of plaque based on the Grey-Weale score, plaque size and estimation of plaque grey-scale median (PGSM). cIMT was measured in diastole from the best quality images from each of the 3 angles over the distal 1 cm length of each common carotid artery. The intima-media thickness in the internal and external carotid arteries was not assessed, in keeping with guideline recommendations [26]. The mean cIMT from the far walls of right and left common carotid arteries (mean−mean) was calculated. Carotid plaque was defined as the presence of focal wall thickening at least 50 % greater than that of the surrounding vessel wall or as a focal region with carotid intimal medial thickness (cIMT) > 1.5 mm protruding into the lumen distinct from the adjacent boundary in any location in the extracranial carotid arteries[26].

### 2.3 Neuropsychological assessment

We assessed cognitive function with a standardized neuropsychological test battery that has been validated for cross-cultural settings as previously described [27,28]. We assessed risk of clinically significant global cognitive impairment using the Community Screening Instrument for Dementia (CSI-D)[29], and assessed three cognitive domains: memory (Consortium to Establish a Registry for Alzheimer's Disease (CERAD) total, immediate and delayed), executive function (digit span backward, color trails part A and B) and language (Animal naming, Boston naming test). To generate a composite score for the CSI-D, a standard algorithm was applied [30]. Raw neuropsychological test scores were standardized into *z*-scores for each ethnic group and averaged to create scores for specific cognitive domains, an exploratory principal components analysis having previously established that there was a single-factor solution for each cognitive domain in each ethnic group (data not shown). To prevent fatigue, trained staff conducted cognitive evaluations that lasted roughly 30 min early on the day of clinic attendance. Depression was assessed using the ten-item Geriatric Depression Scale (GDS).

### 2.4 Neuroimaging sequences and analysis

All subjects underwent MRI examination at University College Hospital using a 3T MRI system (Philips Achieva, Eindhoven, Netherlands) with an 8 channel phased array head coil, following a previously described protocol [31]. The imaging protocol included a sagittal T1-weighted 3D TFE sequence with specific parameters (TR/TE/TI 7/3.2/836 ms, flip-angle 18, voxel size 1mm^3^). Cortical grey matter was automatically parcellated into lobes using Geodesic Information Flows (GIF), and these outputs were utilized in the BaMoS algorithm for automatic WMH segmentation [31]. The BaMoS algorithm employed unsupervised hierarchical model selection, enabling accurate identification and delineation of WMH.

Brain tissue segmentation was performed using the GIF algorithm, involving cortical grey matter parcellation and erasure of the signal for skull matter, [32]. The methodology included a multi-atlas segmentation technique, STEPS (Similarity and Truth Estimation for Propagated Segmentations), for segmenting various brain structures, including hippocampal volumes.

Non-periventricular lesion classification, referring to lesions not extending beyond 25 % of the ventricular border, was automatically conducted by assessing their confluency level. An overall coalescence score (CS) was computed, by considering the ratio of lesions in each confluency category relative to the total WMH volume. CS provides a metric for evaluating the overall clustering and merging of WMH within the brain [34].

### 2.5 Statistical analyses

Analyses were performed with Stata v.18.0 (StataCorp, College Station, TX, USA). Continuous data describing the sample were summarised as means and standard deviations (SD) or median (interquartile range) for skewed data, and categorical data as counts and percentages. Normality was assessed through frequency histograms, QQ plots and Shapiro-Wilk tests. Generalized linear regression modelling (GLM) was used to examine the association between carotid atherosclerosis and cognitive or neuroimaging outcomes using an identity link with a Gaussian family distribution, or a log link function and a gamma family distribution as appropriate. In cases where heteroskedasticity assumptions were not met, robust regression was performed using the White-Huber sandwich estimator. Results were summarised as regression coefficients with 95 % confidence intervals (CI). The number of plaques was modelled in two ways: including people with no plaque coded as zero (# plaque + 0) or excluding people without plaque (# plaque).

Two adjusted models were used: 1) minimal adjustment for age, sex, and ethnicity; 2) full confounder adjustment i.e., model 1 plus education, physical activity category, BMI, HTN, diabetes, total and HDL cholesterol, atrial fibrillation, smoking, existing CVD (i.e. coronary heart disease, stroke, or heart failure), alcohol consumption category, presence of chronic kidney disease and GDS. Confounders were chosen using a directed acyclic graph informed by prior knowledge. Models including brain or WMH volume were also adjusted for total intracranial volume. The possibility of effect modification by sex was examined in all models by including a sex interaction term; if this was not statistically significant (P < 0.05) both sexes were pooled for analysis. In all cases there was no evidence of modification by sex so only pooled data are presented. Assumptions for GLM were checked and the possibility of nonlinear relationships was investigated using fractional polynomials but there was no convincing evidence in any model for non-linear relationships so these were not analysed further. Analyses were performed using complete case analysis, which is valid under the assumption that missingness was independent of outcomes. Statistical inference was based on a combination of p-values, effect sizes and CI.

## 3 Results

Participant characteristics are shown in Table 1. The average age was 73 years and 56 % of participants were male. The ethnicity of participants was 44 % European, 33 % South Asian and 23 % African Caribbean. 24 % of participants had diabetes and 59 % had hypertension.

**Table 1:** Characteristics of participants.

Variables	N	Mean/%	SD [IQR]
Age, y	985	73.2	6.7
Male sex	553	56 %	
Ethnicity			
European	437	44 %	
South Asian	326	33 %	
African Caribbean	228	23 %	
Systolic blood pressure, mmHg	985	141.1	18.1
Diastolic blood pressure, mmHg	985	79.3	10.7
Heart rate, bpm	985	67.4	11.4
Height, cm	984	165.3	9.0
Body mass index, kg/m^2^	985	27.9	4.7
Fat percent	957	32.5	8.2
Total cholesterol, mmol/l	962	4.6	1.1
High density lipoprotein cholesterol, mmol/l	962	1.5	0.4
Triglycerides, mmol/l	962	1.3	[0.7]
Glycated haemoglobin, mmol/mol	953	40.5	9.6
Diabetes mellitus	242	24.4 %	
Coronary Heart Disease	124	13.1 %	
Stroke	15	1.6 %	
Hypertension	568	58.9 %	
Chronic kidney disease	161	16.8 %	
Urinary albumin: creatinine ratio	954	0.06	0.01
Alcohol consumption			
None	373	37.6 %	
≤14 units per week	539	54.3 %	
>14 units per week	80	8.1 %	
Physical activity category			
Low	236	32.07 %	
Moderate	261	35.46 %	
Moderate to high	193	26.22 %	
High	46	6.25 %	
Current smoker	27	2.7 %	
Presence of any carotid plaques	144	14.5 %	
Right side	87		
Left side	88		
Plaque area, mm^2^	126	38.6	26.9
Plaque Grayscale Median	126	84.0	31.4
Carotid intima-media thickness, mm	928	0.89	0.21
People with carotid stenosis.	50	5 %	

### 3.1 Carotid atherosclerosis and cognitive function

A comparison of cognitive function by presence or absence of plaque or carotid stenosis is shown in Fig. 1a, Fig. 1b, Fig. 1c, Fig. 1da–d. CSID (a measure of global cognitive function), memory, executive function and language performance were lower in people with carotid plaques. Fig. 1a Comparison of neuroimaging biomarkers by the presence or absence of plaque is shown in Fig. 1b. WMH lesion volume and CS were lower in people with plaque. When people with and without carotid stenosis >50 % were compared, only executive function was convincingly lower in those with carotid stenosis and no differences in neuroimaging biomarkers were observed, but the limited number of people with carotid stenosis meant that estimates were imprecise. Fig. 1c & Fig. 1d

**Fig. 1a:**
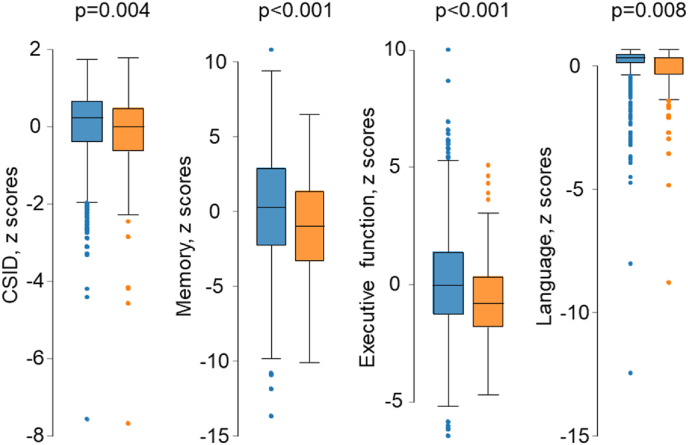
Comparison of cognitive function by presence or absence of plaque. People with plaques (orange) and without (blue). Abbreviations: CSID – Community Screening Instrument for Dementia. (For interpretation of the references to color in this figure legend, the reader is referred to the Web version of this article.)

**Fig. 1b:**
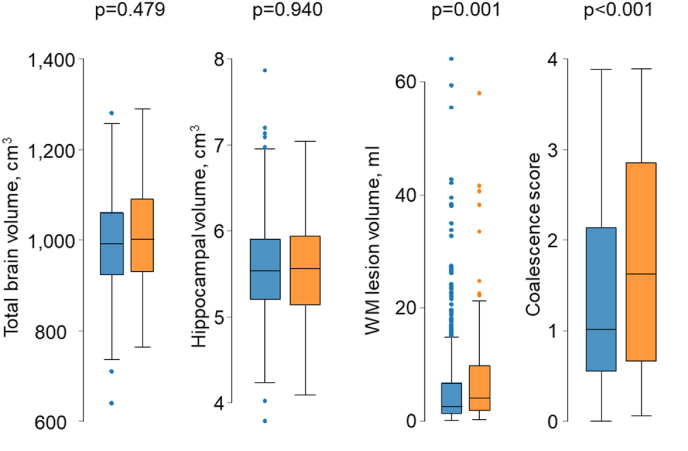
Comparison of neuroimaging biomarkers by presence or absence of plaque. People with plaques (orange) and without (blue). WM – white matter. (For interpretation of the references to color in this figure legend, the reader is referred to the Web version of this article.)

**Fig. 1c:**
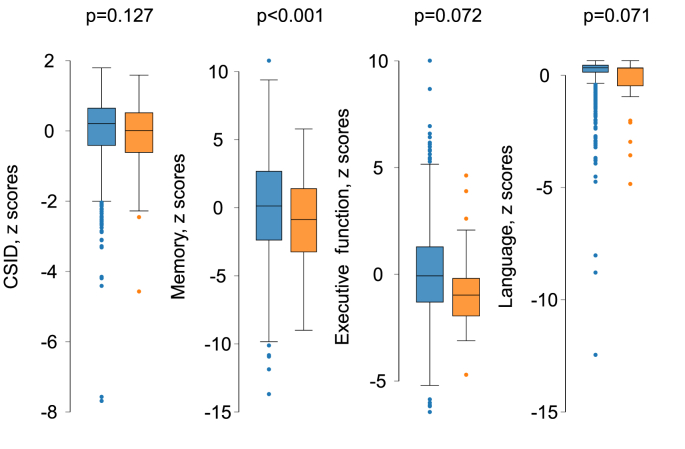
Comparison of cognitive function by presence or absence of stenosis >50 %. People with plaques (orange) and without (blue). Abbreviations: CSID – Community Screening Instrument for Dementia. (For interpretation of the references to color in this figure legend, the reader is referred to the Web version of this article.)

**Fig. 1d:**
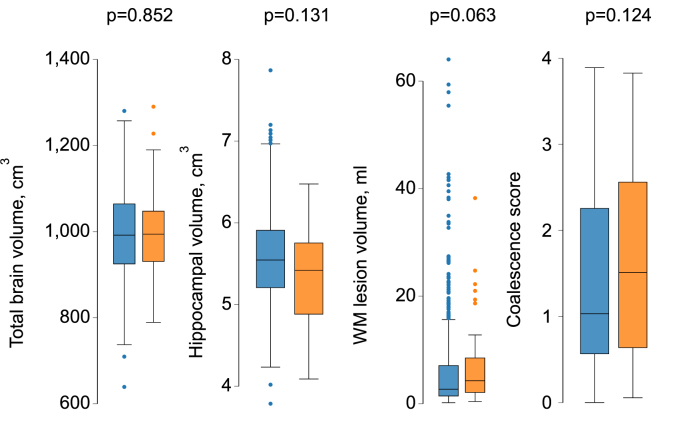
Comparison of neuroimaging biomarkers by presence or absence of stenosis >50 %. People with plaques (orange) and without (blue). WM – white matter. (For interpretation of the references to color in this figure legend, the reader is referred to the Web version of this article.)

Table 2 presents the results of a rank correlation analysis between cIMT, plaque characteristics and cognitive functional domains. There was evidence of an association between cIMT, plaque area and #plaques+0 and CSID but no other convincing associations with CSID. When examining specific cognitive domains, for memory and executive function, negative correlations were observed with cIMT, plaque area, and #plaques+0. There was no convincing evidence of correlations between language and any of cIMT or plaque characteristics except plaque area and #plaques+0.

**Table 2:** Spearman correlation coefficients (*r*_*s*_) between cIMT, plaque characteristics and cognitive functional domains.

Cognitive domain	cIMT, mm	Plaque area, mm^2^	#plaques+0	#plaques	Minimum PGSM	Class (manual)	Class (auto)
CSID	r_s_ = -0.071 p = 0.032	r_s_ = -0.084 p = 0.009	r_s_ =-0.086 p = 0.008	r_s_ = 0.109 p = 0.241	r_s_ = −0.021 p = 0.819	r_s_ = −0.053 p = 0.590	r_s_ = 0.037 p = 0.713
Memory	r_s_ = -0.120 p = 0.003	r_s_ = -0.110 p <0.001	r_s_ =-0.111 p < 0.001	r_s_ = 0.167 p = 0.075	r_s_ = 0.058 p = 0.532	r_s_ = −0.168 p = 0.096	r_s_ = −0.011 p = 0.902
Executive function/attention	r_s_ = -0.097 p = 0.003	r_s_ =-0.128 p <0.001	r_s_ =-0.127 p < 0.001	r_s_ = 0.120 p = 0.206	r_s_ = 0.043 p = 0.666	r_s_ = −0.157 p = 0.149	r_s_ = −0.029 p = 0.756
Language	r_s_ = -0.052 p = 0.875	r_s_ = 0.082 p = 0.011	r_s_ = -0.083 p = 0.011	r_s_ = 0.096 p = 0.306	r_s_ = -0.013 p = 0.886	r_s_ = -0.179 p = 0.079	r_s_ = -0.053 p = 0.563

**Fig. 2:**
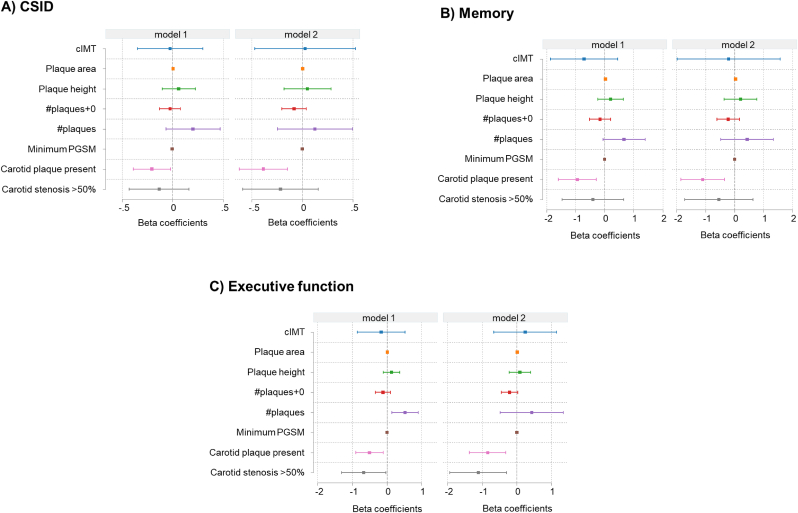
**(A**–**C).** Forest plots summarising the association between measures of carotid atherosclerosis and cognitive function measures (A) CSID, B) memory, C) executive function) after adjustment for age, sex ethnicity (model 1), and model 1 plus education, physical activity category, BMI, HTN, diabetes, total and HDL cholesterol, atrial fibrillation, smoking, existing CVD (i.e. coronary heart disease, stroke, or heart failure), alcohol consumption category, presence of chronic kidney disease and depression (model 2).

Results after adjustment for potential confounders are shown in Fig. 2 (A-C). The presence of carotid plaque was independently associated with poorer CSID, memory and executive function/attention, and carotid stenosis was associated with poorer executive function after full adjustment (model 2). All other associations between cognitive metrics and carotid atherosclerosis measures were attenuated after adjustment, such that none of the association were convincingly different from null. No convincing associations were observed with language and most attenuation following adjustment was attributable to age (data not shown).

### 3.2 Carotid atherosclerosis and neuroimaging findings

Table 3 shows rank correlations between cIMT and plaque characteristics with total brain volume, hippocampal volume and WM lesion volume and CS. There were no correlations between cIMT or plaque characteristics with total brain volume or hippocampal volume. cIMT, #plaque+0 and PGSM correlated positively with WM lesion volume. cIMT, #plaque+0 were also positively associated with CS.

**Table 3:** Spearman correlation coefficients (*r*_*s*_) between cIMT/plaque characteristics and brain volumes and WMH measurements.

Neuroimaging measure	cIMT, mm	Plaque area, mm^2^	#plaques+0	#plaques	Minimum PGSM	Class (manual)	Class (auto)
Total brain volume	r_s_ = 0.004 p = 0.899	r_s_ = 0.144 p = 0.156	r_s_ = 0.036 p = 0.336	r_s_ = 0.093 p = 0.362	r_s_ = −0.013 p = 0.893	r_s_ = −0.230 p = 0.308	r_s_ = 0.100 p = 0.323
Hippocampal volume	r_s_ = −0.068 p = 0.065	r_s_ = −0.060 p = 0.553	r_s_ = −0.004 p = 0.915	r_s_ = −0.038 p = 0.709	r_s_ = −0.149 p = 0.146	r_s_ = −0.182 p = 0.110	r_s_ = −0.177 p = 0.080
WM lesion volume	r_s_ = 0.082 p = 0.026	r_s_ = −0.088 p = 0.393	r_s_ = 0.100 p = 0.006	r_s_ = −0.133 p = 0.197	rs = 0.295 p = 0.021	r_s_ = 0.040 p = 0.614	r_s_ = −0.073 p = 0.463
Coalescence score	r_s_ = 0.097 p = 0.014	r_s_ = −0.144 p = 0.157	r_s_ = 0.100 p = 0.008	r_s_ = −0.100 p = 0.353	r_s_ = 0.193 p = 0.520	r_s_ = 0.013 p = 0.899	r_s_ = −0.054 p = 0.503

**Fig. 3:**
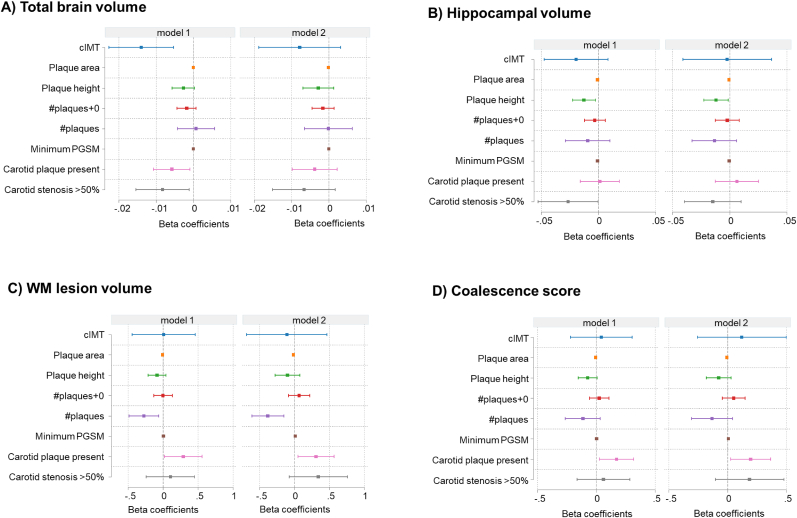
**(A**–**D).** Forest plots summarising the associations between measures of carotid atherosclerosis and neuroimaging measures (A) Total brain volume, B) Hippocampal volume, C) WM lesion volume, D) Coalescence score) after adjustment for age, sex ethnicity (model 1) and model 1 plus education, physical activity category, BMI, HTN, diabetes, total and HDL cholesterol, atrial fibrillation, smoking, existing CVD (i.e. coronary heart disease, stroke, or heart failure), alcohol consumption category, presence of chronic kidney disease and depression.

Associations between cIMT or plaque measures and neuroimaging measures after adjustment are shown in Fig. 3(A-D). Presence of plaque or carotid stenosis was associated with lower total brain volume after adjustment for age and sex, ethnicity and TIV, but full adjustment for confounders attenuated this association. Similarly higher cIMT was associated with lower total brain volume following adjustment for age, sex, ethnicity and TIV, but full adjustment for confounders attenuated this relationship. There were no convincing associations between other plaque characteristics and total brain volume in either model. With the exception of plaque height which was negatively associated with hippocampal volume, there were no convincing associations between presence of plaque, carotid stenosis, cIMT or plaque characteristics with hippocampal volume. Presence of plaque was associated with higher WM lesion volume and CS in both models (Fig. 3C & D). #plaques was associated with lower WM lesion volume, no other convincing associations were observed.

## 4 Discussion

We investigated associations between measures of carotid atherosclerosis and cognitive performance and neuroimaging measures of brain health in a multi-ethnic population cohort. In unadjusted analyses, several measures of carotid atherosclerosis were associated with poorer cognition, including impaired executive function and memory, but following adjustment for potential confounders, particularly age, most associations except for associations with presence of plaque were substantially attenuated or abolished. Presence of plaque was associated with increased WM lesion volume and increased WM lesion coalescence, but there was no convincing evidence of associations between cIMT and any neuroimaging measure of brain health once confounding had been accounted for. The more robust relationship between plaque and various outcomes may indicate that plaque is a more definitive indicator of carotid atherosclerosis than cIMT, which in part may reflect an adaptive response to local transmural pressure[35,36]

Our findings can be compared with previous research examining associations between cognitive impairment and carotid atherosclerosis. A study conducted by Auperin et al. [37] did not identify any statistically significant correlations between carotid atherosclerosis and cognitive function in women; however, associations were observed between carotid plaque and mini-mental state examination scores (MMSE) and Digit Symbol Substitution Test (DSST) scores in men. The Cardiovascular Health Study [38] found that presence of plaque was associated with lower performance on MMSE and DSST, but that this relationship was attenuated when accounting for risk factors related to vascular disease. Similarly correlations between cIMT and decreased MMSE and DSST scores were weakened when vascular risk factors were taken into consideration. In another study of individuals diagnosed with cardiovascular disease [39] higher carotid intima-media thickness (cIMT) was observed to be associated with poorer memory, but no correlations were observed in relation to processing or executive function; however, this study did not fully account for potentially confounding risk factors. Whitehall II [40] reported that cIMT showed no discernible correlation with cognitive state and memory. Another study [41] also found no correlation between carotid plaque burden and cognition. Variability in study samples, cognitive testing methods and severity of carotid atherosclerosis probably contribute to some differences between studies. Another potential source of inter-study difference is the extent of cognitive reserve [42]. The neural implementation of cognition involves two key components: neural reserve, reflecting inter-individual differences in cognitive processing within a healthy brain, and neural compensation, encompassing adaptive changes in cognitive processing to address the challenges posed by brain pathology. Together, these components contribute to an individual's capacity to maintain cognitive function in the presence of neurological damage or pathology [43,44] these components may mitigate effects of cerebrovascular disease and underly differential associations between the extent of cerebrovascular disease in an individual and its functional consequences. Cognitive reserve is also likely to differ between different populations, and this may contribute to differences between studies.

We observed that carotid plaques were linked to lower total brain volume in line with prior studies[45] but this relationship was attenuated after full adjustment for risk factors. We also observed associations between presence of carotid plaque and WM lesion volume that were independent of risk factors. This is consistent with the findings of a systematic review and meta-analysis [46], which observed a 42 % increased probability of WMH in individuals with carotid atherosclerosis in comparison to those without; however, Moroni et al., noted that the association between carotid atherosclerosis and brain outcomes could be attributable to shared risk factors that were not accounted for in their analysis. Our findings suggest that associations with presence of plaque cannot be fully accounted for by measured confounders. Another study [47] also observed no association between common carotid cIMT and WMH, total brain volume or hippocampal volume after adjustment for risk factors, although the intima-media thickness of the internal carotid artery was associated with WMH and total brain volume. In the Northern Manhattan study [48,49] an intima-media thickness that was a composite of the intima media thickness of the common carotid artery, the bulb and the internal carotid was associated with WMH volume even after adjusting for risk factors; however they reported that associations for cIMT were not significant after adjustment. In our study of older people, the internal carotid artery was infrequently visualised, and it was not possible to measure the intima-media thickness of the internal carotid artery reliably. It seems plausible that differences in method of assessment of carotid artery intima-media thickness (composite intima-media thickness vs. cIMT), could contribute to differences between studies. The other factor that could contribute to between study differences is varying degrees of cognitive reserve as discussed above.

Our study has strengths and limitations. Strengths include that it: 1) uses a multi-ethnic population-based sample that includes the two major ethnic minorities in the UK; 2) carotid atherosclerosis was measured using standardised ultrasound protocols, which included assessment of plaque characteristics including PGSM and echogenicity; 3) cognition was assessed using domain-specific cognitive tests that have been validated in ethnic minority participants; 4) detailed brain imaging and analysis was performed; and comprehensive adjustment for possible confounders was possible due to the detailed phenotyping that was undertaken during the clinic visit. Limitations include cohort attrition which may have introduced bias and reduced the representativeness of the sample, the possible presence of residual or unmeasured confounding despite extensive phenotyping, the use of multiple comparisons which increases the likelihood of false discovery, and the cross-sectional design which precludes causal inference.

## 5 Conclusions

This cross-sectional study of a multi-ethnic cohort of individuals resident in UK, provides evidence that carotid that presence of carotid plaque, is associated with poorer cognitive function and brain health. Associations with cIMT or plaque characteristics were unconvincing once confounding had been accounted for.

## Author contributions

RA, AH, and MR designed the study. RA performed data analysis. RA and SS prepared the first draft of the manuscript. AH, NC & MR supervised the project. All authors critically reviewed the manuscript for intellectual content. All authors had final responsibility for the decision to submit for publication.

## Funding

RA receives support from 10.13039/100019236Department of Radiological Science, 10.13039/501100008406Faculty of Applied Medical Sciences, 10.13039/501100004054King Abdul-Aziz University, Jeddah, Saudi Arabia. AH receives support from the 10.13039/501100000274British Heart Foundation, the Horizon Programme of the European Union, the National Institute for Health Research University College London Hospitals Biomedical Research Centre, the UK Medical Research Council, and the 10.13039/100010269Wellcome Trust. NC, AH, and MR work in a unit that receives support from the UK Medical Research Council. BCCP received infrastructure support from the National Institute for Health Research University College London Hospitals Biomedical Research Centre and the 10.13039/501100000274British Heart Foundation.

## Declaration of Competing interest

The authors declare that the research was conducted in the absence of any commercial or financial relationships that could be construed as a potential conflict of interest.

Abbreviations: cIMT, carotid intima media thickness; CSID, Community Screening Instrument for Dementia; #plaques, numbers of plaques in only people with plaque; #plaques+0, number of plaques in all participants (i.e. includes those without plaque coded as zero); PGSM, plaque grey-scale median.
